# Predicting the efficacy of microwave ablation of benign thyroid nodules from ultrasound images using deep convolutional neural networks

**DOI:** 10.1186/s12911-025-02989-7

**Published:** 2025-04-11

**Authors:** Enock Adjei Agyekum, Yu-guo Wang, Eliasu Issaka, Yong-zhen Ren, Gongxun Tan, Xiangjun Shen, Xiao-qin Qian

**Affiliations:** 1 Department of Ultrasound, Affiliated People’s Hospital of Jiangsu University, Zhenjiang, 212002 China; 2 School of Computer Science and Communication Engineering, Jiangsu University, Zhenjiang, Jiangsu Province China; 3 Department of Ultrasound, Jiangsu Hospital of Integrated Traditional Chinese and Western Medicine, Nanjing, China; 4 College of Engineering, Birmingham City University, Birmingham, B4 7XG UK; 5 Northern Jiangsu People’s Hospital Affiliated to Yangzhou University, Yangzhou, China; 6 Northern Jiangsu People’s Hospital, Yangzhou, Jiangsu Province China; 7 The Yangzhou Clinical Medical College of Xuzhou Medical University, Yangzhou, Jiangsu Province China

**Keywords:** Thermal ablation, Benign thyroid nodules, Convolutional neural networks, Deep learning, Volume reduction rate

## Abstract

**Background:**

Thyroid nodules are frequent in clinical settings, and their diagnosis in adults is growing, with some persons experiencing symptoms. Ultrasound-guided thermal ablation can shrink nodules and alleviate discomfort. Because the degree and rate of lesion absorption vary greatly between individuals, there is no reliable model for predicting the therapeutic efficacy of thermal ablation.

**Methods:**

Five convolutional neural network models including VGG19, Resnet 50, EfficientNetB1, EfficientNetB0, and InceptionV3, pre-trained with ImageNet, were compared for predicting the efficacy of ultrasound-guided microwave ablation (MWA) for benign thyroid nodules using ultrasound data. The patients were randomly assigned to one of two data sets: training (70%) or validation (30%). Accuracy, sensitivity, specificity, positive predictive value, negative predictive value, and area under the curve (AUC) were all used to assess predictive performance.

**Results:**

In the validation set, fine-tuned EfficientNetB1 performed best, with an AUC of 0.85 and an ACC of 0.79.

**Conclusions:**

The study found that our deep learning model accurately predicts nodules with VRR < 50% after a single MWA session. Indeed, when thermal therapies compete with surgery, anticipating which nodules will be poor responders provides useful information that may assist physicians and patients determine whether thermal ablation or surgery is the preferable option. This was a preliminary study of deep learning, with a gap in actual clinical applications. As a result, more in-depth study should be undertaken to develop deep-learning models that can better help clinics. Prospective studies are expected to generate high-quality evidence and improve clinical performance in subsequent research.

## Key points

Thermal ablation presents a less invasive approach for addressing thyroid nodules.

Deep learning can predict the efficacy of microwave ablation in patients with thyroid nodules.

The volume reduction rate holds significant importance in the application of microwave ablation

## Background

Thyroid nodules are common in clinical settings, and their detection in adults has improved due to the use of ultrasound (US) [1]. Benign thyroid nodules can induce subjective symptoms or cosmetic issues that are related to nodular volume [1]. Large thyroid nodules can induce local pain and swallowing difficulties [2] due to esophageal and tracheal compression [3], necessitating surgery. While the majority of thyroid nodules are not malignant, surgery remains the preferred therapeutic option [3–5]. Thyroidectomy and surgery are the standard, first-line therapies for benign and malignant thyroid nodules, according to the 2015 American Thyroid Association guidelines and the 2016 Chinese expert consensus and guidelines [5]. Patients frequently refuse surgery because of the related complications, such as permanent scarring, recurrent laryngeal nerve injury, and long-term dependency on levothyroxine [5, 6].

US-guided thermal ablation (TA), which includes methods such as microwave ablation (MWA), laser ablation (LA), and radiofrequency ablation (RFA), is a less invasive approach that has been shown to shrink nodules and preserve thyroid function [7] and is recommended in relevant treatment guidelines [8–12]. They work by generating tissue necrosis with heat and are less expensive than traditional treatments like surgery [13–15]. The volume reduction ratio (VRR) is a direct metric for assessing the clinical efficacy of ablation [16–18]. Individuals experience varying degrees and rates of lesion absorption following ablation. Various factors, including the morphology of the nodules, may impact the effectiveness of US-guided percutaneous TA. Moreover, as indicated in literature reports, between 5% to 30% of patients with nodules experience unsatisfactory outcomes, characterized by a VRR below 50% following initial TA and experience nodule regrowth [19–23]. Additionally, effective predictive models that analyze the factors influencing the efficacy of benign thyroid nodule ablation are limited. Nodules that decrease to a lesser degree are more prone to recurrence [24]. If thermal therapies are viewed as a viable alternative to surgery, then VRR becomes an important factor in their adoption [25]. Being able to predict the efficacy of thermal treatments could help identify the best candidates for a successful outcome in a single session. Nodules that do not respond adequately will be removed surgically or treated further.

US imaging is chosen over other medical imaging modalities as the initial screening test because it can often reveal thyroid abnormalities [26, 27]. Sometimes it’s hard to tell apart the overlapping texture patterns of the thyroid tumors in the US images, even for an experienced radiologist [28, 29]. Deep learning (DL) approaches can be used to train a model to recognize statistical patterns, classifications, and data-driven predictions [30–33]. A subset of these techniques, known as convolutional neural networks (CNNs), has increased in prominence.

CNNs improve early disease detection by enhancing feature extraction, making early stage identification more sensitive. They do detailed assessments using multimodal inputs, such as images, and employ transfer learning to perform better with less data [34, 35]. Real-time image processing is now possible due to recent advances in computational efficiency, which has accelerated diagnosis. CNNs outperform conventional approaches in image-based applications by automatically learning hierarchical features and capturing complicated patterns, resulting in enhanced sensitivity and accuracy [34, 35]. They are less susceptible to differences in image quality since they require little preprocessing and are computationally efficient due to parameter sharing. Medical imaging modalities have been thoroughly studied with pre-trained DL networks [33–37]. The DL image characterization is split into two groups: pre-trained models and self-design models [33, 38].

Pre-trained models are frequently used to illustrate transfer learning techniques. Transfer learning begins by utilizing patterns discovered while solving a specific problem, as opposed to beginning from scratch [33, 39]. CNN is getting more and more popular because of its enhanced performance and simplicity of training. In a number of image vision tasks, it has shown encouraging results [33–37, 40]. Two prior studies demonstrated that classical machine learning models based on clinical and US features of benign thyroid nodules may predict RFA and MWA efficacy [25, 41], and help clinicians develop appropriate treatment strategies. In the preceding investigations, clinical characteristics and US features were first extracted, and feature selection was performed before developing the models. The extraction of features and feature selection are time-consuming and difficult. The extracted and selected features affect the performance of the classification if not chosen judicially. The DL approach excludes the process of manual feature extraction.

In this study, we aimed to develop and assess CNN algorithms based on transfer learning utilizing US images of benign thyroid lesions to predict the efficacy of MWA in patients with benign thyroid nodules before surgery. To our knowledge, no research has been undertaken on predicting the efficacy of MWA for patients with benign thyroid nodules utilizing DL models, specifically CNN. We trained our model directly on the US images without first extracting features, which we hope physicians would comprehend and utilize.

## Methods

### Patients

**Fig. 1:**
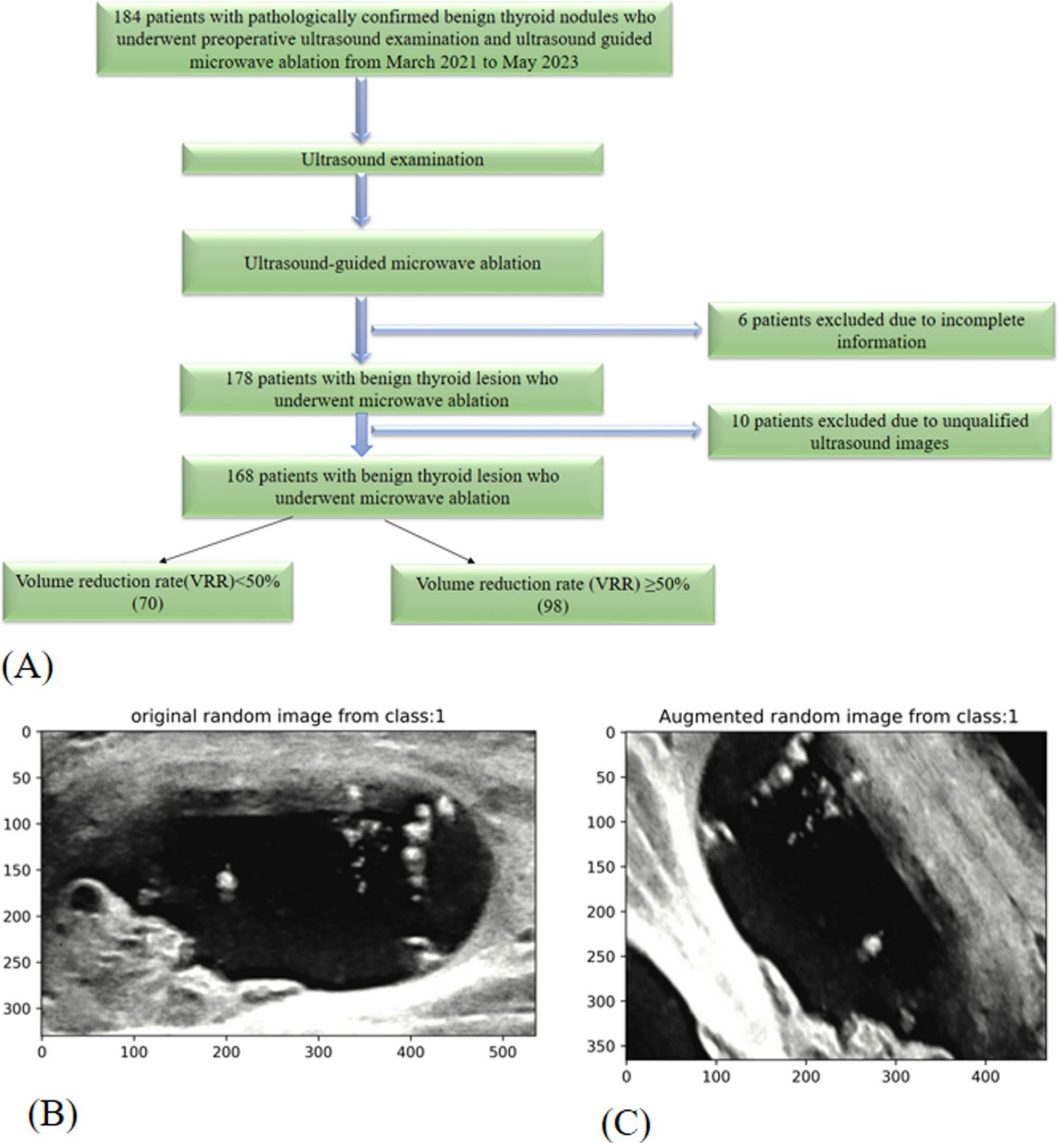
(**A**) Schematic diagram of the patient selection. (**B**) An original random image (**C**) an augmented random image. VRR, volume reduction rate

From March 2021 to May 2023, a retrospective selection was conducted on 168 patients at Jiangsu Hospital of Integrated Traditional Chinese and Western Medicine. The enrolment process is illustrated in Fig. 1A. All patients underwent routine 2-dimensional US examinations and US-guided MWA. Based on the 3-month follow-up examination results post-US-guided MWA, which served as the ground truth, cases were categorized into VRR ≥ 50% and VRR < 50% groups. The patients were randomly assigned to one of two data sets: training (70% n = 118 patients with 184 US images) or validation (30% n = 50 patients with 80 US images). Inclusion and exclusion criteria are detailed in Table 1. This retrospective study was approved by Jiangsu Hospital of Integrated Traditional Chinese and Western Medicine.

**Table 1:** Inclusion and exclusion criteria

Inclusion and exclusion criteria
Inclusion criteria 1. Routine US with complete images and clear quality, and the US image is typical benign, the pathological result of fine needle aspiration cytology is benign, and the BRAF gene test result of puncture fluid is also negative 2. Patients did not receive chemoradiotherapy or other cancer treatment prior to ablation
Exclusion criteria 1. Pathology results that cannot be identified as benign 2. Unclear images with incomplete nodules 3. Pregnant and lactating women 4. Patients with a severe allergic history or severe cardiopulmonary disease

US; ultrasound

### Ultrasound examination

Before MWA, all patients had a routine US examination performed by well-trained technicians using a Philips Q5, Philips iU22 (both Healthcare, Eindhoven, the Netherlands) or a GE LOGIQ s8, LOGIQ E20, LOGIQ E9 (GE Medical Systems, American General) US system with a 5–12 MHz linear array transducer. The patients were placed in the supine position, and longitudinal and transverse continuous scanning were performed to obtain longitudinal and transverse images of the thyroid nodules. This enabled the measurement of thyroid tumor initial volume, shape (regular or irregular), nodule type (cystic, solid, mixed, predominantly cystic), internal echo pattern (even or uneven), and tumor boundary (clear, unclear). The target nodules were identified, and images of the maximum transverse and longitudinal sections of thyroid nodules were saved.

### Ultrasound-guided MWA procedure

The MWA device comprised a microwave generator, a flexible coaxial cable with low loss, and an internally cooled antenna akin to a 14 G to 16 G needle. Operating at 2450 MHz, the generator could emit 1–100 W of power either in pulse or continuous mode. This energy caused water molecules within the targeted tissue to oscillate, raising its temperature to cytotoxic levels, and resulting in cell death. Subsequently, the induced coagulative necrosis is degraded by the patient’s immune system. When deciding on the best puncture path and ablation, we considered the size, location, and vascularity of thyroid cystic nodules.

Patients were positioned supine with the neck extended during the procedure. To mitigate pain from needle puncture, lidocaine (2%) (5 ml) was administered into the skin puncture site and thyroid capsule before ablation. Before performing MWA on cystic lesions, the fluid inside the nodules was removed using a 20 ml syringe. The cystic fluid was then mixed with normal saline solution (0.9%) to facilitate suction. MWA was conducted after the nodule had largely depleted its fluid content and had significantly shrunk in size. In this procedure, known as the “barrier” technique, a mixture of normal saline and sodium hyaluronate was injected between the thyroid capsule and the surrounding tissues. This effectively separated the thyroid capsule from the adjacent tissues, creating a liquid barrier zone approximately 5–10 mm wide. This step aims to prevent inadvertent thermal damage to vital neighboring structures such as the laryngeal nerve, carotid artery, and trachea. Additionally, the “artery-first” and “marginal venous ablation” techniques were employed. It is crucial to prioritize ablating the feeding artery to restrict the tumor’s blood supply. Draining veins are commonly present around the periphery of thyroid nodules, which during MWA, contribute to a heat-sink effect, impeding complete ablation of the nodule’s borders.

To tackle this concern, the marginal vein was treated by ablation after its perforation by the electrode tip. This approach reduces bleeding during ablation and mitigates the risk of incomplete ablation of the tumor caused by the heat sink effect. This effect is characterized by adjacent arteries draining thermal energy away from the targeted tissues. The microwave needle was precisely positioned within the nodules with the guidance of US. The MWA treatment commenced with a power output ranging from 25 to 40 W. The ablation process was conducted under dynamic US monitoring. Special attention was paid to the region of the capsule wall and the solid portion of the nodules during MWA. Before and immediately after US-guided MWA, contrast-enhanced US (CEUS) was performed. The lesion was deemed completely ablated when the defected area covered the edge of the primary lesion by 1–2 mm, the surrounding thyroid parenchyma was filled with a contrast agent, and multi-angle scanning revealed a significant change in echo post-ablation. After treatment, all patients were observed for over two hours while receiving a 30-minute local neck compression.

### Follow-up after ultrasound-guided MWA

US assessments were conducted before and three months after ablation. Measurements of nodules’ size and echogenicity were performed, and their volume was calculated. The largest diameter of each nodule was noted (a), along with the other two perpendicular diameters (b and c). Nodule volume was computed using the formula: V = πabc/6. The VRR was determined using the following formula:

$$VRR(\% ) = \frac{{\left( {pretreatmentvolume} \right) - \left( {followupvolume} \right)}}{{\left( {pretreatmentvolume} \right)}} \times 100\% $$

### Data preprocessing and data augmentation

The imaging repository was reviewed to locate all thyroid US images, and the outcomes of MWA were correlated with the US imaging data. Before pre-processing, grayscale conversion was applied to all B-mode US images to eliminate unnecessary image channels. In this study, the region of interest (ROI) of the primary thyroid lesion for each US image was segmented by a radiologist with over 8 years of thyroid US examination experience. The rectangular ROIs were cropped from raw US images according to the tumor segmentation mask, resized to 224 × 224 pixels, and normalized. Previous research has demonstrated the benefits of this frame selection strategy, which is why it was chosen [42, 43]. There are several benefits of using rectangular cropped ROIs as a frame selection strategy as opposed to traditional methods. First of all, it provides targeted focus, enabling the exact isolation of important features within an image. This targeted strategy reduces computing load by processing only the essential data, which leads to shorter training times and less resource consumption.

Model performance is enhanced by cropped ROIs because they increase learning accuracy in tasks like classification. When sized appropriately, they can maintain contextual relevance, which is beneficial for tasks requiring spatial dependence. They are also adaptable, enabling dynamic alterations in reaction to specific objects of interest. Moreover, cropped ROIs enhance input data, which enhances CNN performance and aids models in focusing on critical areas. Comparably simple to apply, this method can be combined with data augmentation techniques to improve training datasets. Compared to full image analysis, feature extraction, and segmentation techniques, cropped ROIs simplify the preprocessing stage and provide targeted and effective input, improving model performance and computing efficiency in image analysis applications. Contextual information, which may be crucial for interpreting spatial relationships in an image, may be lost when employing rectangular cropped ROIs. The fixed size and shape of ROIs may not be sufficient for objects with irregular shapes, and the procedure relies heavily on the efficacy of ROI selection, which can be subjective and time-consuming. Moreover, the model may not be able to generalize to new data due to overfitting from training on certain ROIs. To avoid insufficient mass extraction and to capture some of the surrounding area near the thyroid lesion, which could yield important information, a pixel border was added around the lesion zone in this study. These images were then employed as input for the DL models.

The training set was utilized to optimize the model’s parameters. Given the limited training data available in this study and the need to mitigate overfitting and sample imbalances, a method called data augmentation was employed [44, 45] (Fig. 1B and C), which meant randomly horizontal and vertical flipping the input image, randomly adjusting the height of an image by an amount of 0.2, randomly adjusting the width of an image by an amount of 0.2, randomly zooming into an image by an amount of 0.2, randomly rotating an image by an amount of 0.2. This process ensures that the model focuses on identifying thyroid lesions amidst potential noise sources [45]. We also utilize pre-trained models to leverage learned features from balanced datasets, which enhances model performance on our datasets. Additionally, all augmented images were resized to 224 × 224 pixels to standardize the scale. This strategy, proven effective, helps prevent network overfitting and the memorization of exact training image details [46].

All preprocessing steps were conducted in Python (version 3.10.12) by using the Keras preprocessing (https://www.tensorflow.org/api_docs/python/tf/keras/preprocessing)

### Model construction

A CNN comprises various layers, such as input and output layers, along with convolution, pooling, and fully connected layers. The input layer receives raw data, while convolution layers extract features through convolution operations. Pooling layers further reduce parameter scale, and fully connected layers amalgamate all features for classification. For constructing models to predict the efficacy of MWA for benign thyroid nodules using US images, CNN architectures including VGG19, ResNet50, EfficientNetB1, EfficientNetB0, and InceptionV3 [47–50] with pre-training on ImageNet (http://www.image-net.org/) were employed in this study

VGG19, ResNet50, EfficientNetB1, EfficientNetB0, and InceptionV3 used in this study are all CNN models, and numerous studies have confirmed their ability to efficiently perform classification tasks using US images [42, 43, 51, 52]. These different pre-trained models were obtained from an open-access library (Keras Applications, available at https://www.tensorflow.org/api_docs/python/tf/keras/applications). Transfer learning was utilized to help fine-tune all weights and biases and reduce training time significantly. In our model, parameters pretrained on the ImageNet dataset were used and after loading that, we used our dataset for retraining. At last, the original classifier for the ImageNet classes was replaced by a binary classifier so that the output was a class probability vector ranging from 0 to 1 as the prediction result for each patient.

**Fig. 2:**
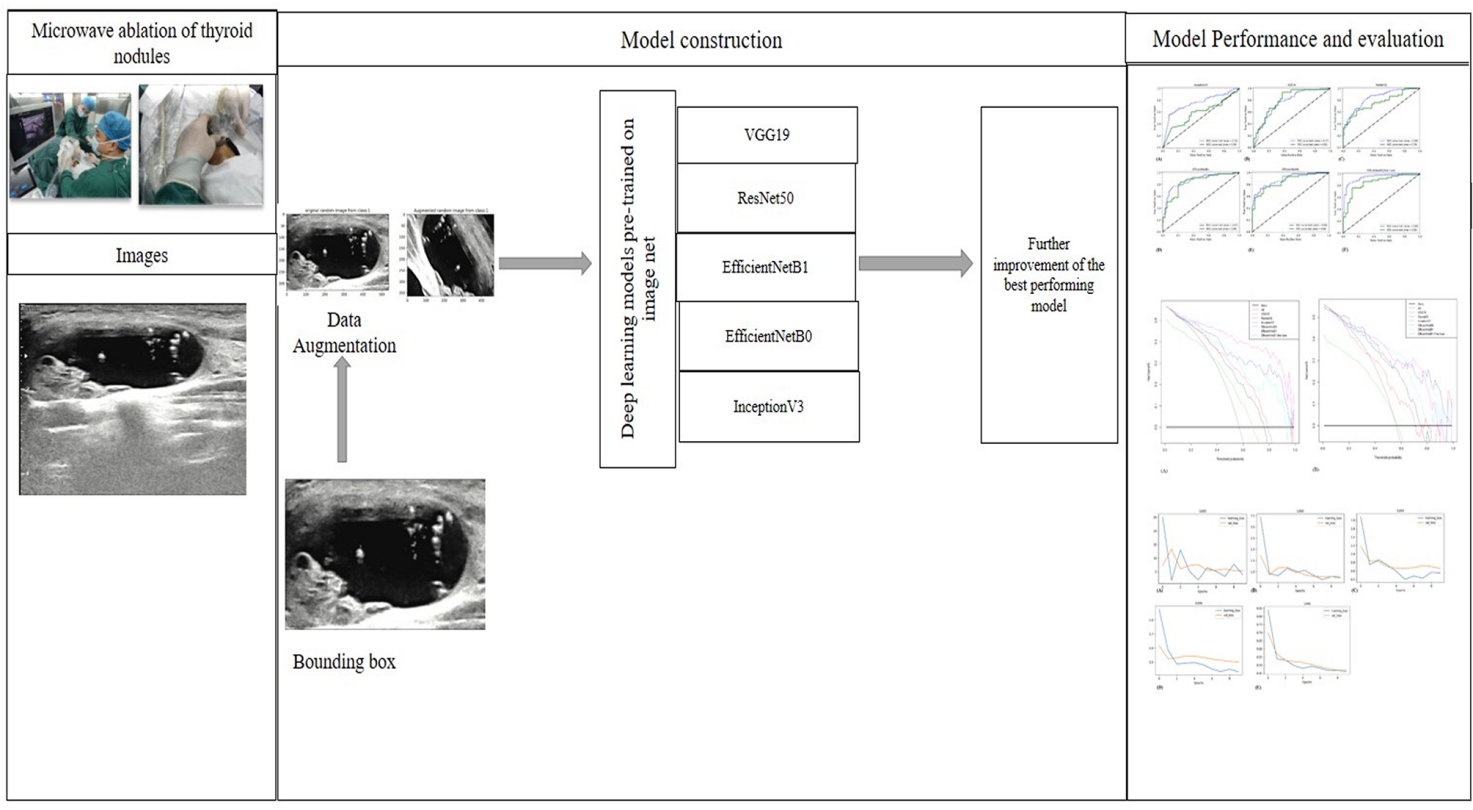
Flowchart of deep learning model construction. Workflow of deep learning model construction for predicting efficacy of thermal ablation in patients with thyroid nodules

The VRR of thyroid lesions post MWA treatment after three months was represented in one-hot encoding as the label. The network was trained from scratch, during the training phase, images of rectangular ROIs were fed into the network as input for updating model parameters via backward propagation. The network’s outputs which is the VRR served as classification results, and the loss function was determined by computing the cross-entropy between the outputs and labels. With a batch size of 32, the Adam optimizer was employed to adjust the model parameters, with a learning rate set at 0.001. We utilized Tensorflow version 2.10.0 and Keras version 2.10.0 for the implementation of the training and validation code. The models underwent 10 epochs of training to prevent overfitting. A flowchart of the study is shown in Fig. 2.

### Statistical analysis

Statistical analysis was conducted utilizing Python (version 3.10.12) and IBM SPSS Statistics for Windows version 26.0 (Armonk, New York, USA). To compare differences in categorical characteristics, either Pearson’s chi-square or Fisher’s exact test was employed. For continuous factors with a normal distribution, the independent sample t-test was utilized, while for those without a normal distribution, the Mann-Whitney U test was applied. A two-sided P-value < 0.05 was considered indicative of statistically significant differences.

The diagnostic capability of the DL models in distinguishing nodules with VRR < 50% or VRR ≥ 50% was demonstrated using the receiver operating characteristic (ROC) curve. This curve plots the true positive rate (sensitivity) against the false positive rate (1 – sensitivity), with the AUC also calculated. Sensitivity, specificity, accuracy, negative predictive value (NPV), and positive predictive value (PPV) of each prediction model were computed using Scikit-learn version 1.2 [53]. DCA was performed using R software (version 3.6.1, https://www.r-project.org).

### Evaluation metrics employed in this study

The models’ performance on both the training and validation datasets was evaluated using metrics such as the AUC, confusion matrix, sensitivity, specificity, NPV, PPV, accuracy, DCA, and other standard clinical statistics. In the prediction of VRR < 50, the confusion matrix provides a concise overview of the model’s performance, juxtaposing its predictions against the real outcomes. The ROC curve plots the true positive rate (sensitivity) against the false positive rate (1 – sensitivity), and the AUC can be calculated. AUC is a metric that is better suited for an imbalance dataset. The accuracy, in the context of VRR < 50% prediction, gauges the model’s overall accuracy in predicting VRR < 50% or VRR ≥ 50% in individuals with thyroid nodules undergoing TA. The accuracy can be calculated from Eq. 1 [54]. Recall or sensitivity (Eqn. 2) for VRR < 50 prediction represents the ratio of correctly predicted cases of VRR < 50 out of all actual cases of VRR < 50% based on the true positive rate, showing a model’s capacity to correctly identify all patients who have VRR < 50% [55].

In the context of VRR < 50% prediction, specificity (Eq. 3) is described in a variety of ways, including a model’s capacity to detect true negatives, being based on the true negative rate, and properly identifying those who do not have VRR < 50%. These evaluation metrics play a crucial role in assessing the efficiency and effectiveness of DL models. In Eqs. 1–5, *TP* represents the number of true positive predictions (correctly predicted positive VRR < 50%), *FP* represents the number of false positive predictions (incorrectly predicted positive VRR < 50%), *TN* represents the number of true negative predictions (correctly predicted negative VRR < 50%), and *FN* represents the number of false negative predictions (incorrectly predicted negative VRR < 50%). These metrics provide valuable insights into the accuracy and performance of VRR predictions.

1 $$accuracy = \frac{{TP + TN}}{{TP + FP + TN + FN}}$$

2 $$sensitivity = \frac{{TP}}{{TP + FN}}$$

3 $$specificity = \frac{{TN}}{{TN + FP}}$$

4 $$PPV = \frac{{TP}}{{TP + FP}}$$

5 $$NPV = \frac{{FN}}{{FN + TN}}$$

The reason we chose this evaluation metrics is because it has proven useful in most clinical research involving the application of artificial intelligence models [41, 42, 56, 57].

## Results

### Clinical characteristics

A total of 168 patients (with 264 US images) with benign thyroid nodules were enrolled, with an average age of 42.26 ± 12.66 years, a range of 13–85 years, and a male-to-female ratio of 43:125. At 3 months following treatment, 98 patients with 150 images (58.3%) achieved VRR ≥ 50%(mean reduction 80%), and 70 patients with 114 images (41.7%) achieved VRR < 50%%(mean reduction 21.39%).

There were 80 patients with solid nodules, with 55 (68.75%) having VRR < 50% and 25 (31.25%) having VRR ≥ 50%. Of the 15 patients with cystic nodules, 7 (46.67%) had VRR < 50%, and 8 (53.3%) had VRR ≥ 50%. Out of 69 individuals with cystic nodules, 7 (10.1%) had VRR < 50%, while 62 (89.9%) had VRR ≥ 50%. There were four patients with mixed nodules, with one (25% of the total) having VRR < 50% and three (75% having VRR ≥ 50%). Most individuals with cystic, predominantly cystic, or mixed nodules had a VRR of ≥ 50%, compared to those with solid nodules. Table 2 displays the clinical data for the VRR ≥ 50% and VRR < 50% groups. The initial volume before ablation, shape, and nodule type (P < 0.05) did differ significantly between the two groups.

**Table 2:** Characteristics of patients and treated nodules divided by 3-month reduction ≥ or < 50%

Clinical Characteristics	VRR < 50%	VRR ≥ 50%	P value
Age	41.77 ± 11.67	46.04 ± 13.09	0.01*
Sex			
Female	54	71	0.31
Male	16	27	
Initial nodule volume, cm^3^	4.93 ± 11.31	10.69 ± 15.21	0.00*
3-month follow-up nodule volume, cm^3^	3.77 ± 7.48	1.78 ± 2.53	0.17
**Nodule type**			0.00*
Solid	55	25	
Cystic	7	8	
Predominantly cystic	7	62	
Mixed	1	3	
**Shape**			0.00*
Regular	35	87	
Irregular	35	11	
**Boundary**			0.59
Clear	66	92	
Unclear	4	6	
**Internal Echo**			0.16
Even	5	13	
Uneven	65	85	

VRR, volume reduction rate, * statistically significant

### Diagnostic performance of the models

**Fig. 3:**
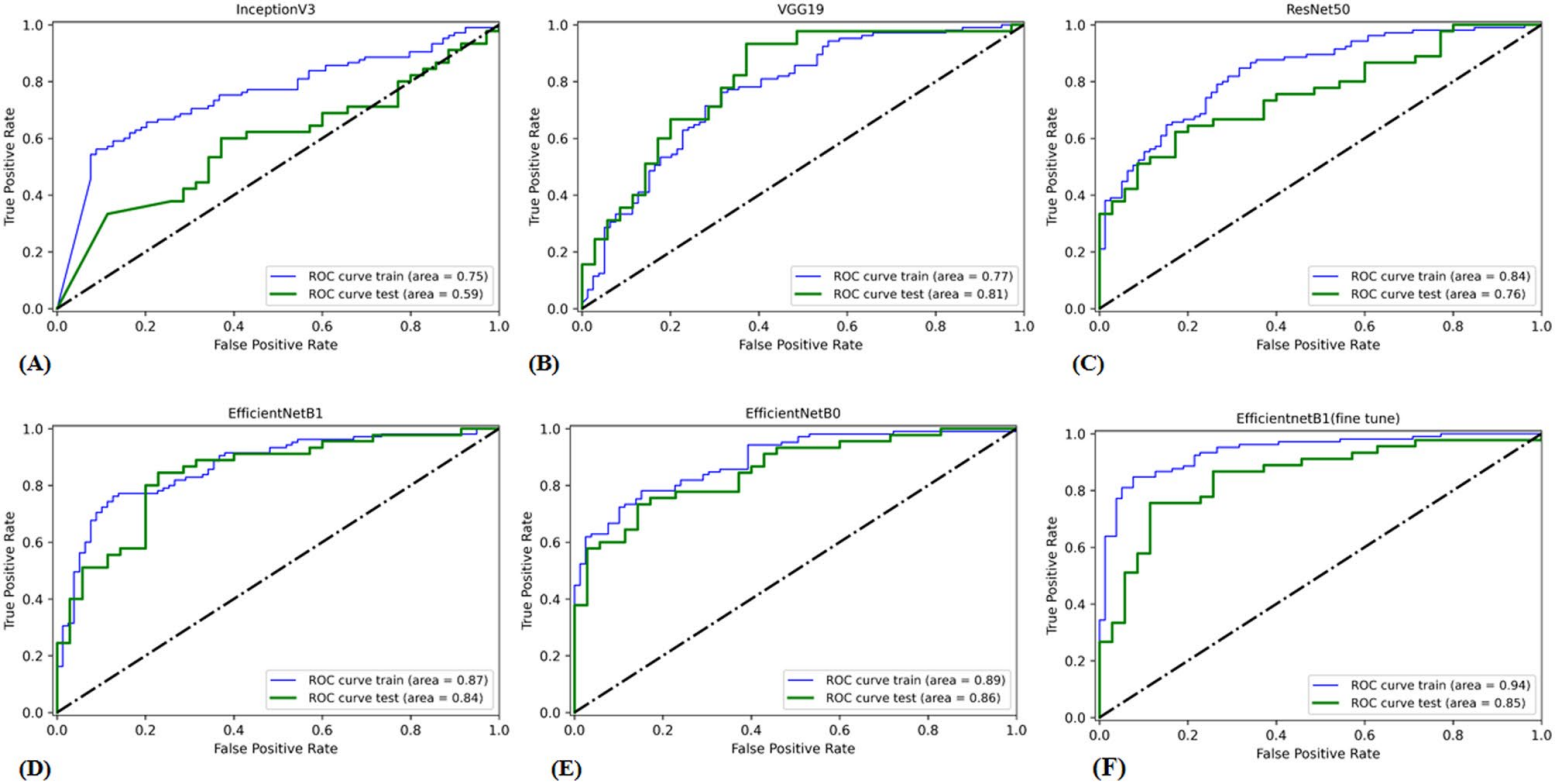
ROC curve of the algorithms used in building the Deep learning models. Green and blue lines represent the validation and train area respectively. ROC, receiver operating characteristic. (**A**) InceptionV3 (**B**) VGG19 (**C**) ResNet50 (**D**) EfficientNetB1 (**E**) EfficientNetB0 (**F**) EfficientNetB1 (fine tune)

**Fig. 4:**
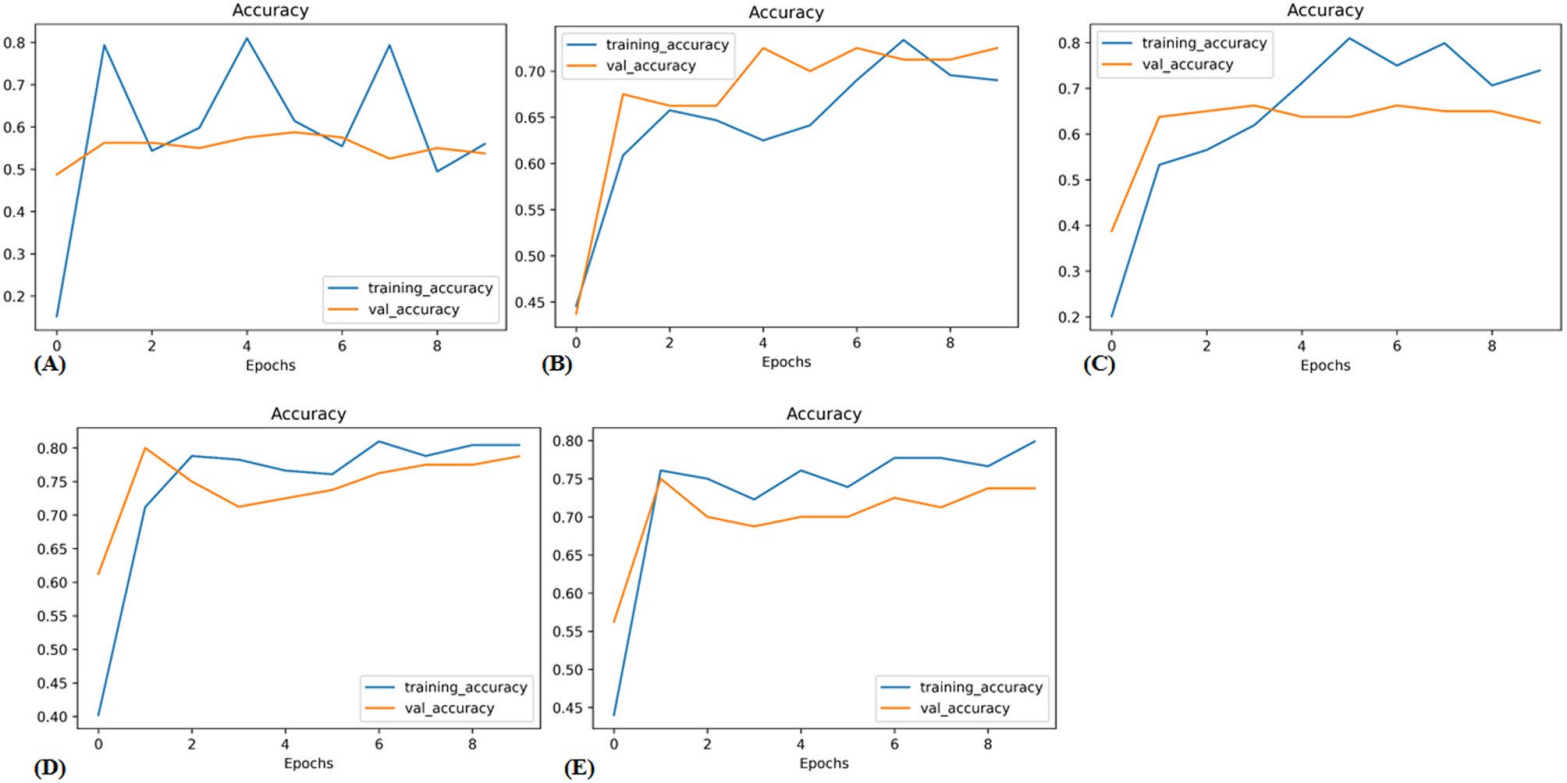
Accuracy curve of the algorithms used in building the deep learning models. Blue and yellow curves represent the training and validation cohort respectively. (**A**) InceptionV3 (**B**) VGG19 (**C**) ResNet50 (**D**) EfficientNetB1 (**E**) EfficientNetB0

**Fig. 5:**
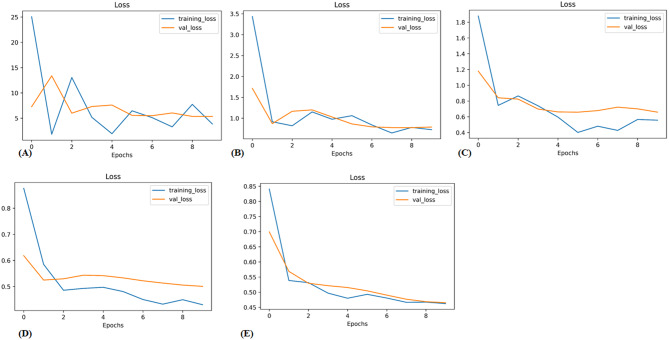
Loss curves of the algorithms used in building the deep learning models. Blue and yellow curves represent the training and validation cohort respectively. (**A**) InceptionV3 (**B**) VGG19 (**C**) ResNet50 (**D**) EfficientNetB1 (**E**) EfficientNetB0

**Fig. 6:**
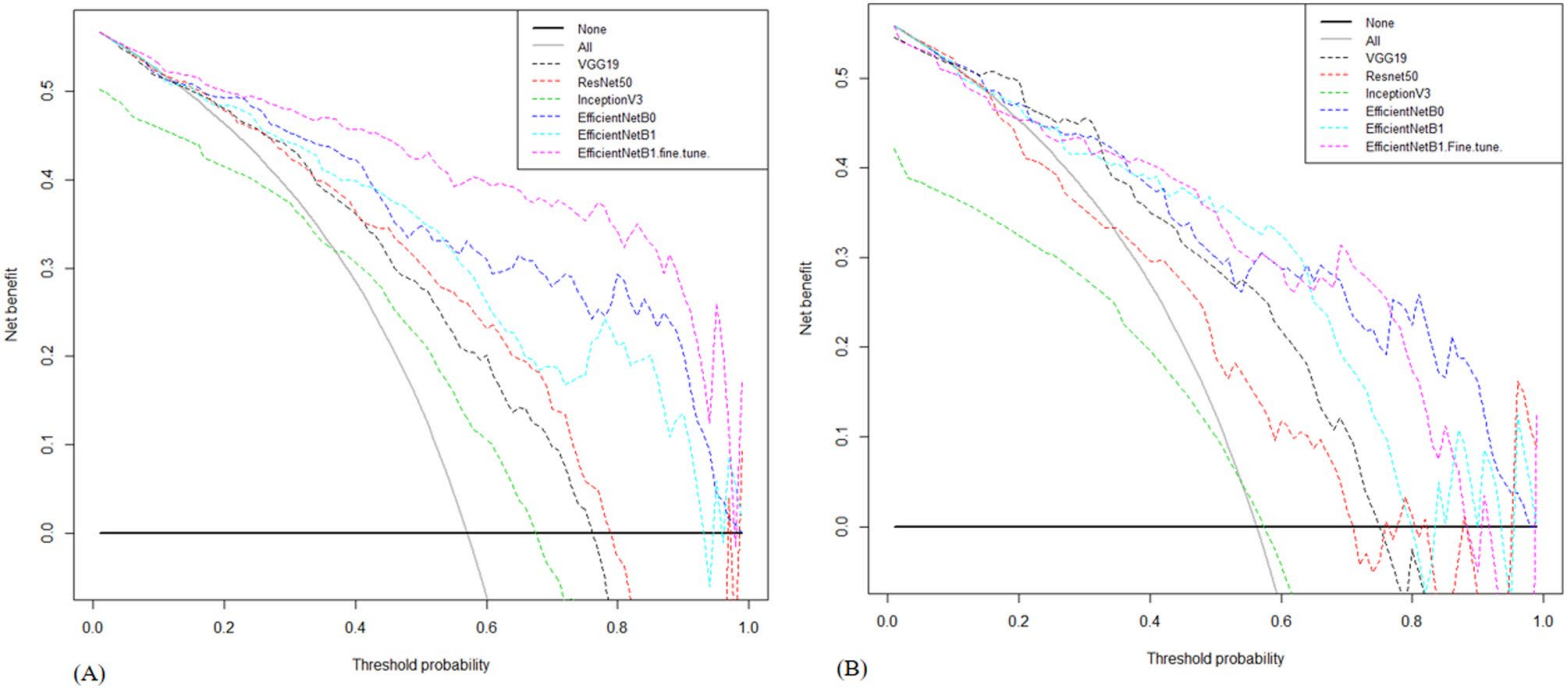
Decision curve analysis of the deep learning models for the training cohort (**A**) and the validation cohort (**B**)

In the validation cohort, AUCs for InceptionV3, ResNet50, VGG19, EfficientNetB0, and EfficientNetB1 were 0.59, 0.76, 0.81, 0.86, and 0.84, respectively. Table 3 displays detailed information about the prediction performance of the DL models. Figure 3 shows the ROC curves of the five DL models. In the validation cohort, the accuracies were 0.54 for the Inception V3 model, 0.63 for the ResNet50 model, 0.73 for the VGG19 model, 0.74 for the EfficientNetB0 model, and 0.79 for the EfficientNetB1 model; the sensitivities were 0.69, 0.80, 0.78, 0.84 and 0.87; and the specificities were 0.34, 0.40, 0.66,0.60, 0.67, respectively. Table 4 displays the confusion matrices depicting the counts of true-positive, false-positive, true-negative, and false-negative outcomes for the classification models. Figures 4 and 5 exhibit the accuracy and loss curves for the five DL models. EfficientNetB1 exhibited superior performance among the five DL models. Additionally, Fig. 6 illustrates the use of DCA to assess the clinical utility of these models.

**Table 3:** Predictive performance of the deep learning models for the training and validation cohorts

	ACC	AUC	SEN	SPEC	PPV	NPV
**Training cohort**						
ResNet 50	0.73	0.84	0.89	0.53	0.72	0.78
Inception v3	0.65	0.75	0.79	0.46	0.66	0.62
VGG19	0.71	0.77	0.86	0.51	0.70	0.73
EfficientNetB0	0.78	0.89	0.86	0.67	0.78	0.78
EfficientNetB1	0.78	0.87	0.90	0.62	0.76	0.83
EfficientNetB1*	0.85	0.93	0.93	0.75	0.83	0.89
**Validation cohort**						
Resnet 50	0.63	0.76	0.80	0.40	0.63	0.61
InceptionV3	0.54	0.59	0.69	0.34	0.57	0.46
VGG19	0.73	0.81	0.78	0.66	0.74	0.70
EfficientNetB0	0.74	0.86	0.84	0.60	0.73	0.75
EfficientNetB1	0.79	0.84	0.87	0.67	0.78	0.80
EfficientNetB1*	0.79	0.85	0.82	0.74	0.80	0.76

AUC, area under the curve; ACC, accuracy; SEN, sensitivity; SPEC, specificity; NPV, negative predictive value; PPV, positive predictive value; * fine-tuned

**Table 4:** Confusion Matrices for the deep learning models

	Inception V3 (Truth)		VGG19 (Truth)		ResNet-50 (Truth)	
Prediction	VRR < 50%	VRR ≥ 50%	VRR < 50%	VRR ≥ 50%	VRR < 50%	VRR ≥ 50%
**Train cohort**						
VRR < 50%	36	22	40	15	42	12
VRR ≥ 50%	43	83	39	90	37	93
**Validation cohort**						
VRR < 50%	12	14	23	10	14	9
VRR ≥ 50%	23	31	12	35	21	36

	EfficientNetB1 (Truth)		EfficientNetB0 (Truth)		EfficientNetb1-fine tune (Truth)	
Prediction	VRR < 50%	VRR ≥ 50%	VRR < 50%	VRR ≥ 50%	VRR < 50%	VRR ≥ 50%
**Train cohort**						
VRR < 50%	49	10	53	15	59	7
VRR ≥ 50%	30	95	26	90	20	98
**Validation cohort**						
VRR < 50%	24	6	21	7	26	8
VRR ≥ 50%	11	39	14	38	9	37

Note.—Data are numbers of images. VRR = volume reduction rate

To further improve the performance of the EfficientNetB1, a small number of layers within EfficientNetB1 were fine-tuned. To adjust the model, its trainable attribute was initially set to true, allowing all previously frozen layers to be unfrozen. Given the relatively limited training dataset, all layers except the last 15 were then frozen again to facilitate training. Subsequently, the model underwent recompilation to integrate these layer modifications. Considering the fine-tuning process, a learning rate 10 times lower (1e-4) was applied to prevent excessive alterations to previously learned weights. The fine-tuned model underwent an additional 10 epochs of training.

**Fig. 7:**
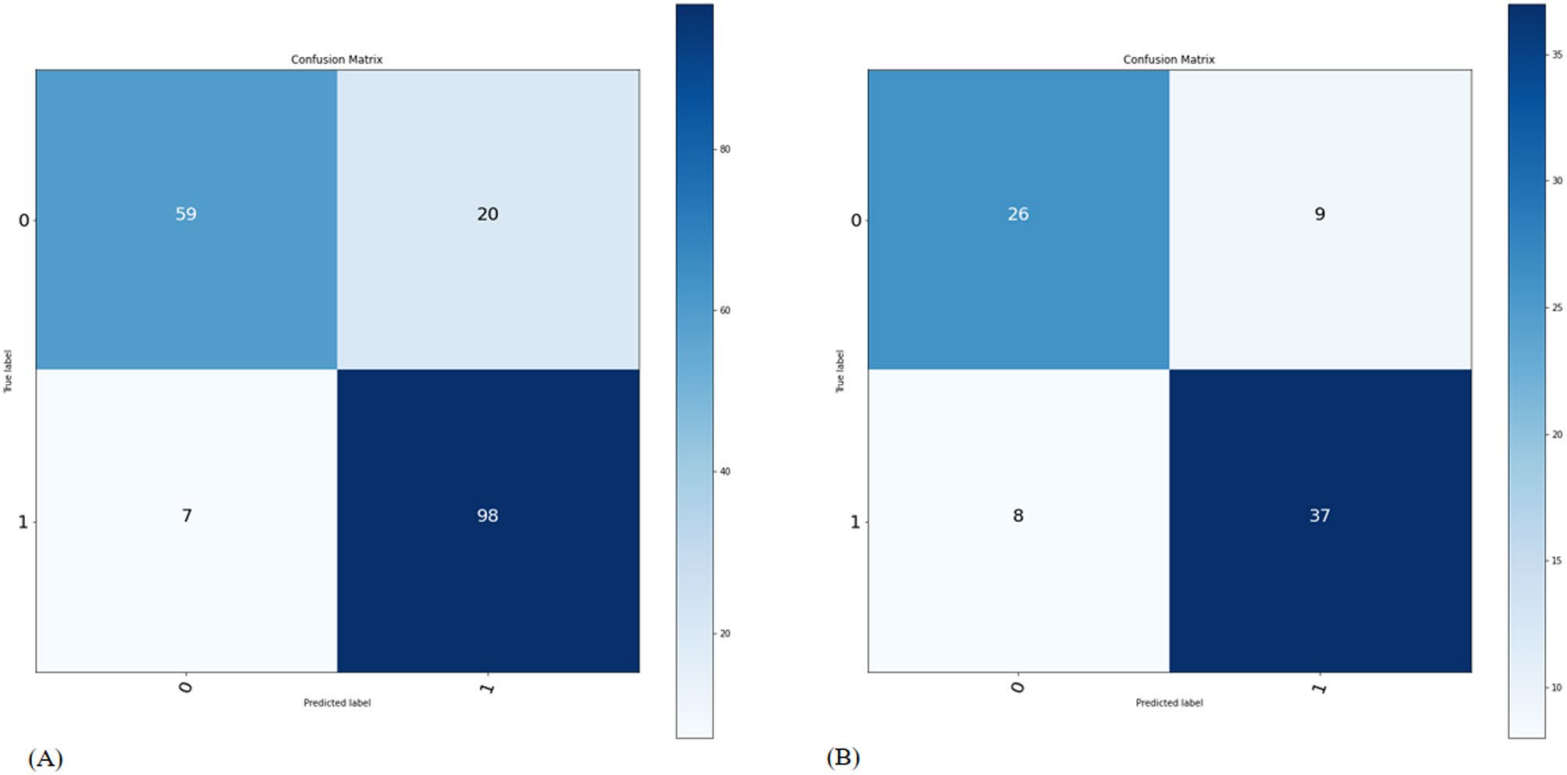
Confusion matrix. The 2 × 2 contingency table reports the number of true positives, false positives, false negatives, and true negatives; training cohort (**A**) and the validation cohort (**B**)

**Fig. 8:**
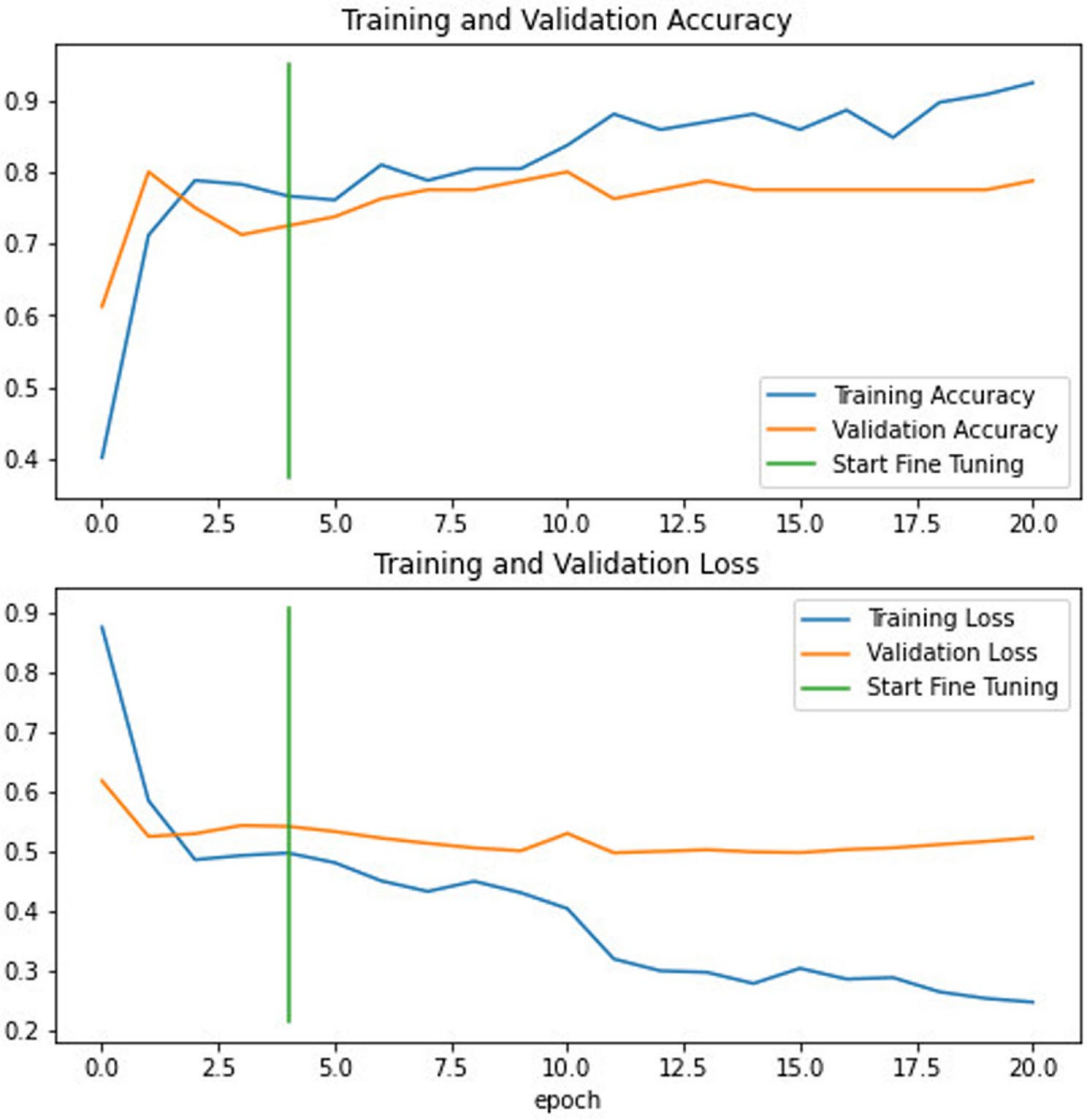
Accuracy and loss curves of EfficientNetB1 model after fine-tuning. Blue and yellow curves represent the training and validation cohorts respectively and the green line represents where fine tune starts

In the validation cohort, the AUC of EfficientNetB1 after fine-tuning was found to be 0.85 (Table 3 and Fig. 3), the accuracy was 0.79; the sensitivity was 0.82; and the specificity was 0.74. The classification confusion matrices that report the number of true-positive, false-positive, true-negative, and false-negative results for the model are shown in Fig. 7 and Table 4. The accuracy and loss trends of the EfficientNetB1 DL model post fine-tuning are depicted in Fig. 8, while the DCA results are illustrated in Fig. 6. Post fine-tuning, the DCA showed that the EfficientNetB1 model yielded substantial overall net benefit, surpassing both the treat-all and treat-none approaches (Fig. 6). Furthermore, compared to InceptionV3, VGG19, ResNet50, EfficientNetB0, and EfficientNetB1, the fine-tuned EfficientNetB1 model demonstrated superior overall net benefit, making it more advantageous than other DL models (Fig. 6).

## Discussion

Patients frequently refuse surgery due to the potential risks. As a result, less invasive methods, such as TA including MWA, are increasingly used to treat thyroid nodules. According to research, ablation techniques carry a lower risk of complications and adverse effects compared to conventional surgical methods [1, 58–62]. When it comes to applying TA, the VRR holds significant importance. In a study involving 104 participants by [60], it was found that after 12 months, 31 individuals (29.8%) exhibited a VRR of less than 50.0%, and 39 patients (37.5%) experienced nodule regrowth. The research indicated that a lower VRR following initial ablation was associated with a higher probability of nodule recurrence.

DL has made significant progress in recent times, facilitating automatic description and understanding of complex data [63–65]. It is worth noting that in this study, VGG19 (ACC: 0.73, AUC: 0.81), ResNet 50(ACC: 0.63; AUC: 0.76), EfficientNetB1 (ACC: 0.79; AUC: 0.84), EfficientNetB0 (ACC: 0.74; AUC: 0.86), and InceptionV3(ACC: 0.54; AUC: 0.59) deep CNNs pre-trained with ImageNet (http://www.image-net.org/) were innovatively tested to determine the best method for obtaining the best results: the EfficientNetB1 DL algorithm performed best in the validation cohort and it was chosen to fine-tune.

The EfficientNetB1’s higher performance in this research may be attributed to its compound scaling, which properly balances depth, width, and resolution. It uses depthwise separable convolutions to minimize parameters while retaining effective feature extraction [49]. The advanced architecture, including squeeze-and-excitation layers, enhances representational power [66], and its pre-training on large datasets aids in generalization for specific tasks [49]. These elements work together to enable the efficient and successful processing of complicated medical images such as medical ultrasonography. The fine-tuned EfficientNetB1 DL algorithm had an ACC of 0.79 and AUC of 0.85, suggesting a good performance for identifying those nodules that will have VRR < or ≥ 50% at 3 months after one MWA treatment session.

DL-based pre-trained models that perform automatic extraction of underlying features provide a more efficient technique of capturing underlying tissue properties for various initial diagnosis and assessments. Our DL model for predicting the efficacy of MWA for benign thyroid nodules is useful for real-time medical systems since it provides rapid diagnostics, delivering results in minutes and reducing turnaround times when compared to traditional methods. With an AUC of 85% and an accuracy of 79%, the model improves diagnostic confidence, perhaps assisting in identifying the most suited patients with thyroid lesions for a successful MWA session. Thyroid nodules that do not respond well will be surgically removed or treated further.

The DL models developed in this study can be readily integrated into existing clinical procedures, hence improving efficiency, by allowing clinicians to obtain data during patient evaluations. Fast identification of thyroid nodules suitable for ablation can be made feasible by this method, which will enable timely interventions which is a crucial component of effective treatment and better patient outcomes. Because of its ability to learn continuously, it can also be updated with new data, ensuring that it remains effective. DL models require less resources to implement and are simpler to utilize; the clinical physician only needs to input the patient’s US image. The method can automatically complete the DL feature extraction to classification process, which is convenient and highly reproducible, easy to promote, and has a promising application. Overall, this DL model can considerably improve the efficiency and effectiveness of thyroid nodule evaluations in real-time medical settings.

Negro et al. [25] used a dataset of 402 cytologically benign thyroid nodules treated with RFA from six Italian institutions to train a machine-learning algorithm. They reported an accuracy of 0.85, but our top-performing DL algorithm had an ACC of 0.79 and an AUC of 0.85. The large patient cohort may have contributed to the study’s higher ACC levels. However, their research focuses on RFA treatment, which is slightly differs from MWA. Also, rather than US images, they focus on predicting VRR < or ≥ 50% using clinical parameters such as baseline nodule volume, echo structure, macro calcifications, and vascularity.

Li et al. [41] employed six machine-learning algorithms to build models that predicted the therapeutic impact of benign thyroid nodule ablation. With an accuracy of 0.79 and an AUC of 0.86, the XGBoost outperformed the others. In comparison to our study, the top-performing DL algorithm, the fine-tuned EfficientnetB1, had an accuracy of 0.79 and an AUC of 0.85, which was consistent with the above study; however, in their study, features were first extracted from the US image before feature selection and model construction. Feature extraction and selection are both time-consuming and complex processes. We trained our model directly on an US image without first extracting features, which we hope clinicians would comprehend and utilize. The DL technique utilized in this study excels at image-based tasks because it automatically learns hierarchical features and captures complicated patterns, improving sensitivity and accuracy. They require little pre-processing and are computationally efficient due to parameter sharing, making them robust to fluctuations in image quality.

Moreover, the DCA was employed to assess the clinical usefulness of these DL algorithms (Fig. 6). Assuming every patient possesses a VRR < 50%, the solid black line (representing the negative scenario) illustrates that when no patient opts for intervention or treatment, the overall benefit remains at zero. Conversely, the solid grey line (representing the positive scenario) illustrates the net benefits when all patients exhibit a VRR < 50% and undergo treatments or interventions. Based on the prevalence of VRR < 50% among patients subjected to TA, the rational range of thresholds was determined from 0.3 to 0.99. Across the entire spectrum, all DL-based algorithms demonstrated superior net benefits compared to the extreme scenarios (negative and positive lines). Throughout almost the entire range of threshold probabilities, the fine-tuned EfficientNetB1 DL algorithm exhibited the highest net benefit in both the training and validation cohorts (Fig. 6).

In DL, the loss function is crucial for model learning. Its primary aim is to minimize the loss magnitude. If the loss fluctuates instead of diminishing, it suggests the model might not be learning effectively. Additionally, if the loss decreases in the training set but remains stagnant in the validation set, it could signify overfitting. In this study, we used categorical cross-entropy to calculate loss for DL models. Figures 4, 5, and 8 show that in both the training and validation cohorts, accuracy was increasing while loss was decreasing, demonstrating the proficient performance of the DL models.

There are some limitations to the study. To begin with, because this is a retrospective study, case selection bias could have influenced the findings. Furthermore, the DL models for predicting the efficacy of ablation were developed and validated in a single hospital using only grayscale US images. Small sample size and single-center data can reduce model generalizability. Small datasets with limited variability may not accurately reflect the population. Furthermore, single-center data might include biases relating to demographics and clinical procedures, limiting its application in varied situations. The lack of external validation imposes further limitations on the evaluation of performance in various settings. These elements cast doubt on predictive models’ clinical usefulness in practical settings. In the future, a multicenter study with a larger sample size is intended. In future investigations, we plan to look at how CNN model’s predictions differ in accuracy between various nodule types.

## Conclusion

The development of DL-based algorithms has attracted much attention for analysing and classification of medical images. A DL model was developed to predict the efficacy of benign thyroid nodule ablation. The present study demonstrates that our DL model is reliable and able to identify nodules that will have VRR < 50% after one session of MWA. Indeed as thermal therapies confronts surgery, predicting which nodules will be poor responders’ represents valuable data that may help physicians and patients decide whether TA or surgery is a better option. This was a preliminary exploration of DL with a gap in actual clinical applications. Therefore, more in-depth research should be conducted to implement DL models that can serve clinics more accurately.

## Data Availability

The original contributions presented in the study are included in the article. Further inquiries can be directed to the corresponding authors.

## Abbreviations

ACC: Accuracy
AI: Artificial intelligence
AUC: Area under the curve
DL: Deep learning
DCA: Decision curve analysis
CNN: Convolutional neural network
LA: Laser ablation
MWA: Microwave ablation
NPV: Negative predictive value
PPV: Positive predictive value
ROI: Regions of interest
ROC: Receiver operating characteristic
SEN: Sensitivity
SPEC: Specificity
TA: Thermal ablation
US: Ultrasound
VRR: Volume reduction rate
